# Dyskerin Overexpression in Human Hepatocellular Carcinoma Is Associated with Advanced Clinical Stage and Poor Patient Prognosis

**DOI:** 10.1371/journal.pone.0043147

**Published:** 2012-08-13

**Authors:** Bei Liu, Jinglei Zhang, Chen Huang, Hui Liu

**Affiliations:** 1 Department of Pathology, Affiliated Tianyou Hospital, Wuhan University of Science and Technology, Wuhan, China; 2 Eastern Hepatobiliary Surgery Hospital, Second Military Medical University, Shanghai, China; 3 Medical Research Center, Peking University Third Hospital, Beijing, China; Mayo Clinic, United States of America

## Abstract

**Background:**

Dyskerin (encoded by the *DKC1* gene) is an essential nucleolar protein involved in cell proliferation, where it is required for the pseudo-uridylation of ribosomal RNA (rRNA) molecules and the stabilization of the telomerase RNA component. Dyskerin expression has been reported to predict poor survival in some cancer patients. The aim of the present study was to analyze the expression of dyskerin in hepatocellular carcinoma (HCC) and to determine its correlation with clinicopathologic features, including the survival of patients with HCC.

**Methodology/Principal Findings:**

Dyskerin protein expression was detected by immunohistochemistry in paraffin sections of 252 HCC cases and 80 noncancerous liver tissues. The correlation was analyzed between dyskerin expression levels and clinicopathologic variables and prognosis. Dyskerin protein was significantly overexpressed in HCC tissues when compared to noncancerous liver tissue. Dyskerin overexpression was positively correlated with the hepatitis B surface antigen status, serum alpha-fetoprotein, and advanced clinical stage in HCC patients. A survival analysis indicated that HCC patients with higher dyskerin expression had a significantly shorter overall survival and 5-year survival time when compared to those with low expression. A multivariate analysis suggested that dyskerin overexpression was an independent factor for prognosis (hazard risk, 2.912; *P* = 0.007). Expression of *DKC1* mRNA was measured by quantitative RT-PCR in 80 HCC and 50 non-cancerous tissues. The relationship between *DKC1*, *TERT*, *MKI67*, and *MYC* mRNA expression in HCC tissues was also evaluated. *DKC1* mRNA was significantly overexpressed in HCC tissues and showed a significant correlation with *MKI67* and *MYC* mRNA but a weak correlation with *TERT* mRNA.

**Conclusions/Significance:**

Dyskerin overexpression in HCC patients was correlated with MYC and MKI67 expression and showed a possible involvement in the tumorigenic process. Dyskerin overexpression may be an unfavorable prognostic factor in patients with HCC.

## Introduction

Hepatocellular carcinoma (HCC) is the fifth most common cancer worldwide and the third most common cause of cancer mortality [1]. The long-term prognosis for the majority of HCC patients remains poor [1], [2]. HCC carcinogenesis is a complex process that can involve various modifications to a number of molecular pathways in addition to genetic alterations, and ultimately leading to malignant transformation and HCC disease progression [3]. The mechanisms underlying the development of HCC remain unclear.

Dyskerin is a nucleolar protein encoded by the *DKC1* gene at Xq28 and is altered in dyskeratosis congenita. The *DKC1* gene is a member of the H/ACA small nucleolar ribonucleoprotein (snoRNP) gene family. Dyskerin is present in small nucleolar ribonucleoprotein particles that convert specific uridine residues of ribosomal (r)RNA to pseudouridine, and it is also a component of the telomerase complex [4]. In humans, functional analyses have revealed multiple roles for dyskerin, including the translational control of specific mRNAs [5], [6], the regulation of a subset of microRNAs [7], [8], and the maintenance of telomere integrity independent of telomere length regulation [9]. Dyskerin is the direct and conserved transcriptional target of c-Myc [10], which explains the strong correlation between its up-regulated expression and active cell proliferation [11].

Increased cell proliferation activity and the rate of cell growth are the important features of neoplasia, which suggests that dyskerin may be intimately involved in malignancies. The overexpression of dyskerin has been reported previously in several cancers, including neuroblastoma [12], lymphoma [13], melanoma [14], breast cancer [15], [16], prostate cancer [17], colorectal cancer [18], [19], and ovarian carcinoma [20]. A previous study also showed that dyskerin is required for tumor cell growth through mechanisms that are independent of its role in telomerase and only partially related to its function in precursor rRNA processing [21].

**Figure 1:**
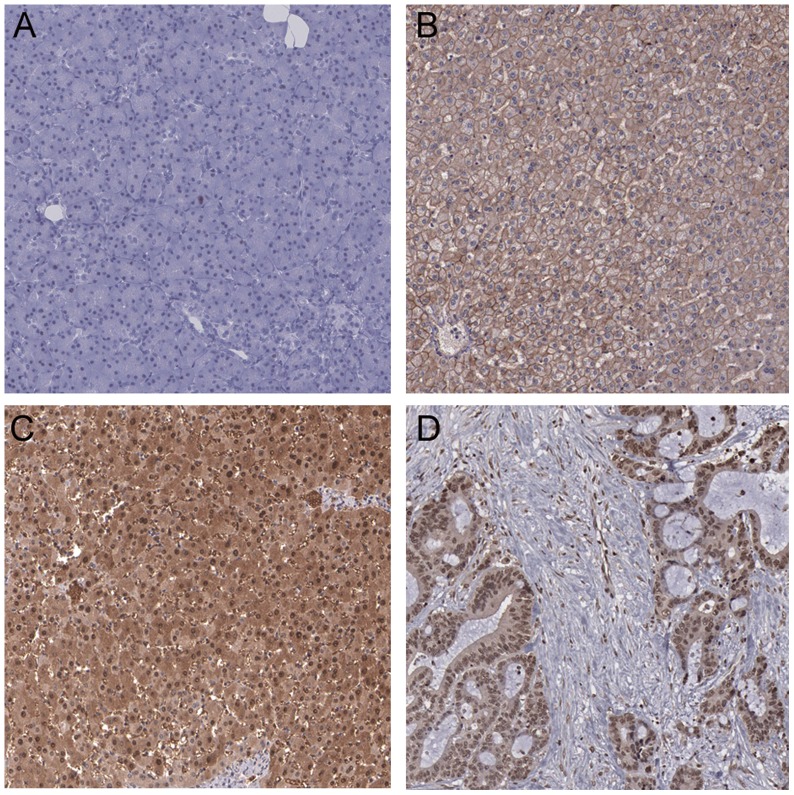
Immunohistochemical analysis of dyskerin protein expression in HCC tissues and noncancerous liver tissues. A. Negative expression of dyskerin in a noncancerous liver sample. B. High expression of dyskerin in a noncancerous liver sample. C & D. High expression of dyskerin in HCC samples.

Although studies have examined the important role of dyskerin in tumorigenesis, research into the role of dyskerin in human HCC is lacking. In this study, our aim was to determine the clinical significance of dyskerin overexpression in HCC tumorigenesis. We analyzed the expression of dyskerin in 252 clinical HCC samples and 80 non-HCC liver samples by immunohistochemistry. We also investigated the relationship between dyskerin expression and the clinicopathologic features and prognosis of patients with HCC. We used real-time RT PCR to evaluate the relationship between the mRNA expression of *DKC1*, human telomerase reverse transcriptase (*TERT*), *MKI67* (encoding the Ki-67 protein), and *MYC* (encoding the c-Myc protein) in HCC tissues. The results suggested that dyskerin overexpression may serve as a marker for the proliferative potential of HCC cells and can be used as an independent marker for the prognosis of patients with HCC.

**Table 1:** Correlations between the clinicopathologic characteristics and dyskerin protein expression in hepatocellular carcinoma.

Characteristics	N	Dyskerin (%)		*P* *
		Negative/low expression	High expression	
Gender				0.55
Male	209	88 (42.1)	121 (57.9)	
Female	43	16 (37.2)	27 (62.8)	
Age (years)				0.36
<60	176	78 (44.3)	98 (55.7)	
> = 60	76	29 (38.2)	47 (61.8)	
HbsAg				0.00
Negative	111	70 (63.1)	41 (36.9)	
Positive	141	58 (43.1)	83 (58.9)	
AFP (ng/ml)				0.00
<400	153	20 (32.0)	104 (68.0)	
> = 400	99	52 (52.5)	47 (47.5)	
Tumor size (cm)^ &^ §				0.81
<5	47	20 (42.6)	27 (57.4)	
> = 5	94	42 (44.7)	52 (55.3)	
Liver cirrhosis §				0.35
Absent	60	26 (44.3)	34 (56.7)	
Mild	51	25 (49.1)	26 (50.9)	
Moderate	54	22 (40.7)	32 (59.3)	
Severe	31	9 (29.0)	22 (71.0)	
Clinical stage §				0.00
I–II	185	102 (55.1)	83 (44.9)	
III–IV	79	24 (30.4)	55 (69.6)	
Adjacent organs invasion §				0.27
Negative	153	78 (51.0)	75 (49.0)	
Positive	74	32 (43.2)	42 (56.8)	
Reccurrence or metastasis §				0.66
Negative	148	57 (38.5)	91 (61.5)	
Positive	76	27 (35.5)	49 (64.5)	
Tumor encapsulation §				0.26
Absent	114	48 (42.1)	66 (57.9)	
Present	109	38 (34.9)	71 (65.1)	

AFP, alpha-fetoprotein; HbsAg, hepatitis B surface antigen.

* : Two-tailed chi-Square test. 0.00 indicates <0.01.

&: Tumor size was measured based on the length of the largest tumor nodule.

§ : Some data were not available, and the statistical analysis was based on the available data.

## Results

### Dyskerin expression was upregulated and associated with several clinicopathologic parameters in HCC

Immunohistochemistry was used to analyze dyskerin expression in paraffin sections obtained from 80 noncancerous liver samples and 252 HCC samples to assess the clinical significance of the dyskerin protein in HCC tumorigenesis. Dyskerin staining was localized mainly in the nuclei of tumor cells and partly in the cytoplasm. Dyskerin expression in noncancerous liver cells was negative or weakly positive, while in HCC tissues, it was moderate or strongly positive (Figure 1). Of the 80 histological non-HCC liver tissues analyzed, 61 tissues (76.3%) showed negative or low dyskerin immunostaining. High dyskerin expression was observed in 148 of 252 (58.7%) HCC tissues, whereas the negative or low expression of dyskerin was observed in 104 (41.3%) of the tissues. These results indicated that dyskerin expression was higher in HCC tissues than in noncancerous liver tissues (*P*<0.01).

The clinicopathological parameters of HCC and their correlations with dyskerin expression are shown in Table 1. High expression of dyskerin showed significant association with hepatitis B surface antigen (HbsAg) status (*P*<0.01), serum alpha-fetoprotein (AFP) levels (*P*<0.01), and advanced tumor stage (*P*<0.01). No significant association was found between dyskerin expression and age, gender, tumor size, cirrhosis, adjacent organ invasion, recurrence, metastasis, or tumor encapsulation.

### Dyskerin overexpression as an independent prognostic marker for HCC

**Figure 2:**
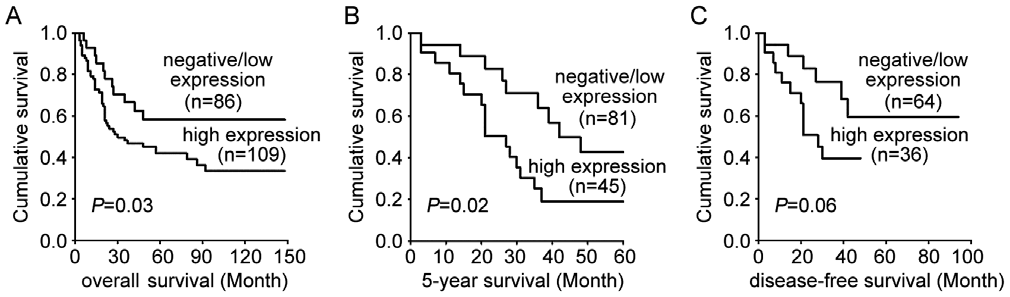
A Kaplan-Meier survival analysis of dyskerin expression in patients with HCC(log-rank test). A. Overall survival of HCC patients: low expression, n = 86; high expression, n = 109. B. Five-year survival of HCC patients: low expression, n = 81; high expression, n = 45. C. Disease-free survival of HCC patients: low expression, n = 64; high expression, n = 36.

Based on the results of the immunohistochemical assay, 195 of 252 HCC patients were divided into a negative/low expression group (n = 86) and a high expression group (n = 109). The correlation between the dyskerin expression level and prognosis was analyzed using the Kaplan-Meier method with the log-rank test. A shorter survival time was found for the high expression group (27.3±3.6 months, median survival time) than for the negative/low expression group (35.6±4.2 months). The difference in the overall survival rate between patients with negative/low and high dyskerin expression was statistically significant (*P* = 0.03) (Figure 2A). High expression of dyskerin was also correlated with a poorer 5-year survival of HCC patients (*P* = 0.02, Figure 2B). Although not statistically significant, dyskerin overexpression was correlated with a shorter disease-free survival time in HCC patients (*P* = 0.06) (Figure 2C).

The possibility that the dyskerin expression level was an independent prognostic factor for the survival of HCC patients was tested by univariate and multivariate Cox regression analyses to identify factors that might predict survival of HCC patients. As shown in Table 2, the univariate Cox regression analysis results indicated that a high dyskerin expression [hazard risk (HR)  = 1.87, 95% confidence interval (CI)  = 1.68–2.54; *P* = 0.02], clinical stage III–IV (HR  = 3.21; 95% CI  = 1.89–4.21; *P* = 0.01), and positive recurrence or metastasis (HR  = 2.76; 95% CI  = 2.19–3.21; *P* = 0.01) were all significantly associated with survival.

**Table 2:** Univariate and multivariate analyses of different prognostic factors with overall survival in 252 patients with HCC (Cox Proportional Hazards Regression).

Parameter	Univariate analysis			Multivariate analysis		
	*P*	HR	95%CI	*P*	HR	95%CI
Age (years)	0.21					
<60		1.0				
> = 60		0.67	0.41–1.02			
Gender	0.37					
Female		1.0				
Male		1.14	0.71–2.36			
HbsAg	0.17					
Negative		1.0				
Positive		1.25	0.94–1.67			
AFP (ng/ml)	0.54					
<400		1.0				
> = 400		1.37	0.62–3.02			
Tumor size (cm) ^&^ §	0.07					
<5		1.0				
> = 5		1.17	0.91–1.51			
Liver cirrhosis §	0.55					
Absent		1.0				
Present		1.23	0.78–2.46			
Clinical stage §	0.01			0.00		
I–II		1.0			1.0	
III–IV		3.21	1.89–4.21		3.16	1.76–3.72
Adjacent organs invasion §	0.08					
Negative		1.0				
Positive		1.48	0.94–1.89			
Recurrence or metastasis §	0.01			0.00		
Negative		1.0			1.0	
Positive		2.76	2.19–3.21		2.75	1.77–3.75
Tumor encapsulation §	0.77					
Absent		1.0				
Present		1.42	0.72–2.81			
Dyskerin expression	0.02			0.00		
Negative/low expression		1.0			1.0	
High expression		1.87	1.68–2.54		2.91	2.84–4.91

AFP, alpha-fetoprotein; HbsAg, hepatitis B surface antigen; HR, hazards ratio; CI, confidence interval.

0.00 indicates <0.01.

&: Tumor size was measured based on the length of the largest tumor nodule.

§ : Some data were not available, and the statistical analysis was based on the available data.

A multivariate Cox regression analysis showed that high expression of dyskerin was an independent risk factor for adverse overall patient survival (HR  = 2.91; 95% CI  = 2.84–4.91, *P*<0.01). Of the other parameters, clinical stage III–IV (HR  = 3.16; 95% CI  = 1.76–3.72, *P*<0.01) and positive recurrence or metastasis (HR  = 2.75; 95% CI  = 1.77–3.75, *P*<0.01) were also revealed as independent prognostic predictors for worsen overall survival in HCC patients.

### Verification of *DKC1* mRNA overexpression by quantitative real-time PCR

**Figure 3:**
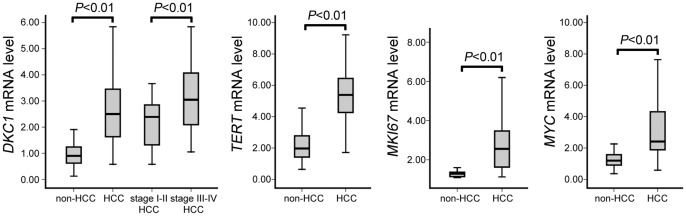
A quantitative real-time PCR analysis of *DKC1*, *TERT*, *MKI67*, and *MYC* mRNA levels in HCC tissues and noncancerous liver tissues. mRNA expression was evaluated relative to the expression of the reference gene *GAPDH*. The data for each sample were averaged from three independent experiments.

The overexpression of *DKC1* mRNA in HCC tissues was further verified by quantitative real-time PCR in the same 80 fresh-frozen HCC samples and 50 fresh-frozen noncancerous liver samples used for the immunohistochemical analysis. As shown in Figure 3, *DKC1* mRNA expression was 2.71-fold higher in the HCC than in the non-HCC liver samples and this difference was statistically significant (*P*<0.01). Moreover, in the cancer group, the level of mRNA in high dyskerin expression cancers was higher than that in low dyskerin cancers (*P*<0.05). A comparison of *DKC1* mRNA expression between 40 stage III–IV HCC tissues and 40 stage I–II HCC tissues revealed a moderate increase in *DKC1* mRNA in stage III–IV HCC tissues (*P*<0.01). These findings further confirmed the overexpression of *DKC1* mRNA in HCC compared to noncancerous liver tissues, particularly for stage III–IV disease.

### Correlation between *DKC1* mRNA expression and *TERT*, *MKI67*, and *MYC* mRNA expression

The correlations between the mRNA expression of *DKC1* and *TERT*, *MKI67*, and *MYC* were evaluated by quantitative real-time PCR for each HCC and noncancerous tissue samples. *TERT*, *MKI67*, and *MYC* mRNA expression was higher in cancer tissues than in non-HCC liver samples (Figure 3). In HCC, *DKC1* mRNA expression was significantly and positively correlated with *MYC* mRNA (Spearman coefficient r = 0.65, *P*<0.01) and moderately correlated with *MKI67* mRNA (Spearman coefficient r = 0.42, *P* = 0.03). A weak association was found between *DKC1* and *TERT* mRNA expression (Spearman coefficient r = 0.21, *P* = 0.07). Overall, *DKC1* mRNA overexpression in HCC tissues was related to the cancer cell proliferation.

## Discussion

The nucleolar protein dyskerin is a core component of the telomerase complex and is required for normal telomere maintenance and the post-transcriptional processing of precursor rRNA [4], [22], [23]. Recent studies have also confirmed that dyskerin overexpression is involved in tumorigenic processes in other cancers [12], [13], [17], [20]. However, the expression of dyskerin and its potential prognostic impact on HCC has not yet been explored. Therefore, the potential role of dyskerin in HCC progression was the focus of the present study.

In the present study, the expression levels of dyskerin protein and mRNA in HCC tissues and adjacent non-malignant liver tissues were examined by immunohistochemistry staining and real-time PCR. Our results established that the protein and mRNA expression of dyskerin was significantly higher in HCC tissues than in non-malignant liver tissues. While this gene has previously been reported to be a tumor suppressor gene [5], [24], the quantitative analysis of dyskerin expression performed on a series of human cancers showed a dramatically increased dyskerin expression in certain types of tumors, including prostate cancer [17], colon cancer [18] and colorectal cancer [19]. Our IHC results demonstrated that the high expression of dyskerin was significantly correlated with advanced clinical stages (III and IV) of HCC. A similar association has also recently been shown between increased dyskerin expression and high clinical stage colon cancer [18] and prostate cancer [17]. Poncet et al. explored the telomeric changes that occur in B-chronic lymphocytic leukemia (B-CLL) and found that the B-CLL cells showed a more than 2-fold decrease in the expression of dyskerin [25]. This diverse expression pattern suggests that the deregulation of dyskerin, together with various gene functions, is involved in different pathogenic mechanisms of human tumorigenesis. The role of dyskerin in the development of hematological malignancies and epithelial cancers requires further research.

We also found that high dyskerin expression was correlated with high AFP and high serum HBsAg levels. Previous studies have indicated that advanced clinical stages and high AFP levels in patients with HCC imply a poor prognosis [26], [27], while HBV infection accounts for the major attributive risk of HCC [28]. Our multivariate Cox regression analyses indicated that high dyskerin expression was an independent risk factor for the prognosis of HCC patients and for clinical stage III–IV and positive recurrence or metastasis. This agrees with previous findings for breast cancer, where low dyskerin expression and activity have been associated with a better prognosis [15]. Therefore, dyskerin appears to play an important role in tumorigenesis and the development of human cancers, including HCC.

Strong cell proliferation activity and anti-apoptosis gene expression signatures are important features of advanced clinical stages of HCC tissues. Increased cell proliferation supported by dyskerin may therefore explain the positive correlation between high dyskerin expression and shorter survival time in HCC patients. Dyskerin is the direct and conserved transcriptional target of c-Myc [10]. In prostate cancer tissues, a moderate and significant correlation has been observed between *DKC1* and *MKI67* mRNA levels, but not with *PCNA* mRNA [17]. In the present study, we noted a significant correlation between dyskerin mRNA expression and *MYC* mRNA expression, and a moderate correlation with *MKI67* mRNA. The transformation of prostate cancer cells with a *DKC1* small interfering RNA resulted in complete cessation of cell growth and proliferation after at most three passages, corresponding to ten days of treatment [17]. These results indicate that the levels of *DKC1* mRNA in HCC tissues are related to the proliferative activity of cancer cells, with a close connection to c-Myc-related transcriptional regulation.

One function of dyskerin is the pseudo-uridylation of rRNA molecules, which is necessary for their processing. A second function is the stabilization of the telomerase RNA component necessary for telomerase activity. However, recent studies indicate that dyskerin contributes to tumor cell growth through mechanisms that do not require the presence of cellular telomerase activity, and which may be only partially dependent upon its role in rRNA processing [11], [29]. In the present study, we found only a weak association between *DKC1* and *TERT* mRNA expression in HCC tissues, consistent with previous reports. This suggests that the molecular role of dyskerin in the modulation of human HCC is yet to be determined.

Dyskerin was first defined as a nucleolar protein that showed strict nucleolar localization. A recent study has indicated that the human *DKC1* gene encodes a new alternatively spliced mRNA that can direct the synthesis of a variant form of dyskerin that has an unexpected cytoplasmic localization [30]. In our study, dyskerin staining was localized mainly in the nuclei of tumor cells and only partly in the cytoplasm. Koskimaa et al. also described dyskerin expression in both the cytoplasm and the nuclei as distinct spots of the basal cells or parabasal cells in cervical lesions tested by immunohistochemistry [31]. Further work is required to learn more about the nucleolar and cytoplasmic forms of dyskerin protein as these might cumulatively account for tumorigenesis.

In summary, the present study confirmed that dyskerin is up-regulated in human HCC tissues and that dyskerin expression is significantly correlated with both the proliferative potential of cancer cells and the advanced clinical stage and prognosis of HCC patients. This suggests that dyskerin may serve as a powerful prognostic marker and therapeutic target for HCC. Future research should focus on understanding its underlying molecular mechanism.

## Materials and Methods

### Tissue samples

A total of 252 HCC specimens and 80 noncancerous liver specimens were obtained as paraffin-embedded samples from the Affiliated Tianyou Hospital of Wuhan University of Science and Technology and the Eastern Hepatobiliary Surgery Hospital, the Second Military Medical University, China. The 252 HCC cases comprised 209 males and 43 females with an age range of 37 to 72 years (median age, 53.4 years). Liver cirrhosis was found in 136 cases (53.9%). The diagnoses were confirmed histologically in all cases, based mainly on the examination of sections stained with hematoxylin and eosin (H&E). Table 1 shows the clinicopathological features of these patients. Tumor stage was determined according to the 2002 International Union Against Cancer TNM classification system [32]. Tumor differentiation was graded by the Edmondson grading system. The documentation of recurrence or metastasis was based on clinical and radiographic findings. Prior consent from patients and approval from the Ethics Committees of the two hospitals were obtained for the use of these clinical materials for research purposes.

### Immunohistochemistry

Immunohistochemical observation was performed using the peroxidase antiperoxidase technique after a microwave antigen-retrieval procedure. The antibody against dyskerin (dilution 1∶100) was obtained from Abcam (Cambridge, UK). This antibody was added to the tissue sections and incubated overnight at 4°C. The secondary antibody (PV-9003 Polymer Detection System, Zhongshan Goldenbridge Biotechnology Co. Ltd., Beijing, China) was added to the sections and incubated at room temperature for 30 minutes and stained with diaminobenzidine (DAB). Images were made at ×200 and digitized, using the same light intensity and color thresholding for all sections of each stain.

### Evaluation of immunohistochemical staining

Two pathologists independently scored the results of the staining. The intensity scoring was defined as follows: 1, no staining; 2, weak staining; 3, moderate staining; and 4, strong staining. The scoring for the number of positive cells was defined as follows: 1, 0–10% positive cells; 2, 10–50% positive cells; 3, 51–75% positive cells; and 4, 76–100% positive cells. The product (score) of the two variables was used to categorize groups with negative/low (score ≤8) and high (score >8) expression.

### RT-PCR and quantitative real-time PCR

Total RNA in tissues previously frozen and stored in liquid nitrogen was extracted using TRIzol Reagent (Invitrogen, Carlsbad, CA). Reverse transcription was performed using a Takara RNA PCR kit (TaKaRa Biotechnology Co., Ltd., Dalian, China) according to the manufacturer's instructions. For quantitative real-time PCR analysis, aliquots of double-stranded cDNA were amplified using a SYBR Green PCR Kit (Applied Biosystems, Foster City, CA) and an ABI PRISM 7900 Sequence Detector. The cycling parameters were 95°C for 30 seconds, 56°C for 20 seconds and 72°C for 1 minute, for 30 cycles, followed by a melting curve analysis. The threshold cycle (CT) was measured during the exponential amplification phase, and the amplification plots were analyzed by SDS 1.9.1 software (Applied Biosystems, Foster City, CA). The primers used were as follows: *DKC1*, forward, 5′-CACTCGCTTGGTGAAGTCACA-3′ and reverse, 5′-CCGGACAATCCCCACATACT-3′; *hTERT*, forward, 5′-TCCACTCCCCACATAGGAATAGTC-3′ and reverse, 5′-TCCTTCTCAGGGTCTCCACCT-3′; *MKI67*, forward, 5′-CCACACTGTGTCGTCGTTTG-3′ and reverse, 5′-CCGTGCGCTTATCCATTCA-3′, and *c-Myc*, forward, 5′-CGTCTCCACACATCAGCACAA-3′ and reverse, 5′-CACTGTCCAACTTGACCCTCTTG-3′. The *GAPDH* gene was used as an internal control, using the following primers: forward 5′-CAAGGCTGTGGGCAAGGT-3′ and reverse, 5′-GGCCATGCCAGTGAGCTT-3′. Each experiment was repeated three times.

### Statistical analyses

All available data in the medical records were recorded into the database. Missing data that were unavailable in the medical records were also noted, and the analyses were based on the available data. All statistical analyses were performed using SPSS 13.0 software. The data were presented as the means ± SDs. The association between dyskerin expression and clinicopathological parameters was evaluated using a chi-Square test for qualitative data and Fisher's exact test for categorical data. Survival curves were plotted using the Kaplan-Meier method and compared using the log-rank test. The significance of different variables with respect to survival was analyzed using the multivariate Cox proportional hazards model. A Mann-Whitney U test was used to compute and compare the difference in dyskerin mRNA expression levels between HCC tissues and non-HCC tissues and between stage I-II HCC tissues and stage III-IV HCC tissues. The correlations between the dyskerin mRNA level and the mRNA levels of TERT, MKI67, and c-Myc were evaluated by Spearman's rank correlation coefficients. A *P* of <0.05 was considered statistically significant.
